# Prevalence and Genetic Variation Investigation of the Pseudorabies Virus in Southwest China

**DOI:** 10.3390/ani14213103

**Published:** 2024-10-28

**Authors:** Jiaqi Wu, Juan Zhang, Jun Zhou, Yi Luo, Xinrong Wang, Rui Yang, Junhai Zhu, Meiyu Jia, Longxiang Zhang, Lizhi Fu, Nan Yan, Yue Wang

**Affiliations:** 1 College of Veterinary Medicine, Southwest University, Chongqing 400715, China; w515610601@126.com (J.W.); juanzh21@163.com (J.Z.); luoyiyl@163.com (Y.L.); wxr13730178925@126.com (X.W.); jamey123123@163.com (M.J.); dyzlx015029@163.com (L.Z.); 2 Sichuan Boce Testing Technology Co., Ltd., Chengdu 610023, China; zhoujun0740@163.com; 3 Chongqing Academy of Animal Science, Chongqing 408599, Chinaflzfulizhi@163.com (L.F.); 4 National Center of Technology Innovation for Pigs, Chongqing 402460, China

**Keywords:** pseudorabies virus, epidemiological survey, genetic variation

## Abstract

In 2022, a significant PRV outbreak in a southwestern China pig farm led to a high incidence of sow abortion. A serological analysis using *gE* antigen-based ELISA revealed a high prevalence (69.30%) of PRV *gE* antibodies among the affected pigs, with a significant variation across different pig populations (1.11–76.12%). We collected additional 5552 pig serum samples and 580 pig cerebrospinal fluid (CSF) samples from various pig farms in Southwest China between 2022 and 2024. The seropositive rates for PRV *gE* antibodies ranged from 2.36% and 8.65% in the serum samples, while the positive detection rates for the PRV *gE* gene in the cerebrospinal fluid samples, as determined by PCR, were between 1.06% and 2.36%. The PCR analysis and sequencing of the PRV *gB*, *gC*, *gE*, and *TK* genes from eight randomly selected samples identified two distinct strains, CQ1 and CQ2. CQ1’s *gC* gene showed similarity to the vaccine strain Bartha, while the other genes aligned with Chinese classical strains, suggesting its potential genetic recombination. CQ2 aligned with the Chinese classical strain SC. Although the overall PRV infection in Southwest China’s pig farms is relatively low, occasional outbreaks with high positivity rates are observed. These findings highlight the necessity for increased surveillance and stringent control measures to safeguard the swine industry.

## Simple Summary

Pseudorabies, a severe acute infectious disease caused by the pseudorabies virus (PRV), is a significant threat to the swine industry, leading to substantial economic losses. This study aimed to assess the prevalence of PRV in southwestern China between 2022 and 2024 to control further spread. Initially, we observed a notably high seropositive rate on a single pig farm, prompting a broader investigation across the region. Fortunately, the overall prevalence of PRV was found to be relatively low, aligning with the patterns observed in other parts of China. We also identified two strains of PRV in tissues of dead pigs. However, the genetic analysis of various PRV genes indicated that the Chinese classical strains remain prevalent, and there is potential for genetic recombination. Consequently, the ongoing surveillance of PRV is essential for an effective disease control and management within the swine industry.

## 1. Introduction

Pseudorabies (PR), also known as Aujeszky’s disease (AD), is caused by PRV, a significant pathogen of the *Alphaherpesvirinae* subfamily within the *Herpesviridae* family. PRV is an enveloped linear double-stranded DNA virus, with a total length of roughly 145 kb, and is recognized for its neurotropic and lymphotropic properties [1,2]. This acute infectious disease can lead to neurological disorders in susceptible animals, with pigs identified as the primary natural reservoir [3]. Moreover, PRV has the capacity to infect a broad range of mammals, including but not limited to mice, cats, foxes, dogs, wolves, and cattle [4,5,6,7,8]. Of particular concern, PRV has been detected and isolated from the cerebrospinal fluid samples of humans [9,10]. The affected patients exhibited respiratory dysfunction and acute neurological symptoms [11,12], indicating the potential for zoonotic transmission. The likelihood of PRV jumping from animals to humans, along with its propensity for mutation and its capacity to co-infect with other pathogens, heightens the complexity of developing effective disease prevention strategies [13].

Clinically, PRV’s impact is age-dependent, with piglets succumbing to severe and debilitating central nervous system disorders post-infection, often proving fatal, with a mortality of 100% [14,15]. In contrast, fattening pigs exhibit respiratory symptoms, while pregnant sows are prone to reproductive disorders and abortion [16]. PRV was first detected in the United States and has since spread to Canada, China, and several other European countries, according to WOAH reports from 2018 to 2024 [17,18]. Many provinces across China have documented the PRV disease, which resulted in substantial economic losses to the swine industry, such as mortality in piglets, abortions in sows, and stunted growth in fattening pigs [14,19]. The introduction of Hungary’s Bartha-K61 *gE*-deleted live vaccine during the late 1970s marked a significant milestone in the prevention and control of PRV [20]. This vaccine, along with gE ELISA serological testing, has played a significant role in managing PRV infections [21]. From the 1990s to late 2011, over 80% of pigs in China were vaccinated with Bartha-K61 [22], significantly enhancing the country’s ability to prevent and control PRV [20]. However, since 2011, the disease has re-emerged in vaccinated farms, posing a renewed and substantial threat to the pig industry in China [23]. The effectiveness of the Bartha-K61 vaccine against PRV has been notable, but the resurgence of the virus emphasizes the need for continuous research in vaccine development. It also highlights the importance of using serological testing for ongoing disease surveillance to track viral adaptations.

The *gE* and *TK* genes of PRV are pivotal in its pathogenicity, with their encoded proteins playing essential roles in disease progression [24,25]. The use of the *gE*-deleted vaccine is now widespread, making the *gE* gene a diagnostic marker to distinguish between pigs naturally infected with PRV and those that are vaccinated [26]. Additionally, the *gB* and *gC* glycoproteins are recognized as key immunogenic proteins that can induce neutralizing antibodies and activate cellular immune responses. The genetic study of these four proteins is fundamental for understanding PRV’s evolution and holds promise for the development of subunit vaccines [27]. Recent research has discovered that the wild PRV strain has the potential to recombine with the *gB* and *gC* genes of the Bartha-K61 vaccine strain, resulting in the emergence of novel recombinant strains with heightened virulence [28]. This recombination event could undermine the effectiveness of conventional vaccinations and challenge the cross-immunity they are designed to provide [29]. The spread of these new PRV variants has been rapid and has caused significant economic losses to China’s swine industry.

Consequently, from 2022 to 2024, we carried out a longitudinal study by collecting samples from a range of pig farms in Southwest China to monitor the prevalence and dissemination of PRV. Concurrently, we conducted a genetic analysis to understand the partial genetic evolution of the virus. The main goal of this study was to enhance our understanding of PRV’s molecular epidemiology, which will be instrumental in developing effective prevention and control strategies tailored to the region.

## 2. Materials and Methods

### 2.1. Sample Collection

In 2022, we conducted a study on a pig farm in Southwest China that had experienced a severe sow abortion incident. We collected 469 serum samples from pigs across different age groups and 6 CSF samples from deceased pigs. To further understand the prevalence of PRV in Southwest China, we expanded our collection to include 3757 blood samples and 283 CSF samples from a diverse range of pig farms across the region over the same year. Continuing our surveillance, between 2023 and 2024, we conducted a comprehensive collection of 1795 serum samples and 297 lumbosacral site puncture CSF samples [30] from pig farms in the region for PRV testing. All the large-scale farms under surveillance adhered to a farrow-to-finish strategy and maintained a regular immunization schedule with the Bartha-K61 live vaccine. Importantly, the collection of samples was carefully timed, taking place within 1–2 days after the initial clinical signs of disease were observed by local veterinarians.

### 2.2. Detection of PRV gE Antibody by ELISA

The serum samples were centrifuged at 13,400× *g* for 10 min to pellet the cells and debris, allowing for the collection of the supernatant for PRV gE-based ELISA analysis. The ELISA was performed in strict accordance with the manufacturer’s instructions for the PRV gE ELISA antibody detection kit (Cat No. MM22801, Manman, Shanghai, China).

### 2.3. Detection of PRV Antigen via PCR

All CSF samples were subjected to viral DNA genome extraction using a viral genomic DNA/RNA kit (Cat No. DP304-02, Tiangen, Beijing, China). Subsequently, specific detection primers for PRV-*gE* were used to amplify and identify the presence of the wild-type PRV pathogen in the samples by detecting the expected amplicon of approximately 250 bp. The amplification was carried out using Takara LA Taq^®^ polymerase with GC Buffer (Cat No. RR02AG, Takara Bio, Beijing, China) to ensure the accuracy of the amplification.

### 2.4. Partial Genome Sequencing

To amplify PRV’s key genes, including *gB*, *gC*, *gE*, and *TK*, we designed specific primers with the Oligo 6.0 software, aligned with the gene sequence of the PRV reference strain (GenBank No. KT809429.1) (Table 1). The *gB* gene was divided into three segments: gB1, gB2, and gB3. Additional primer pairs targeting gE2, gB1, gB2, gB3, gC, and TK were used to amplify the genomic regions encoding these proteins. After amplification, fragments were recovered using a commercial kit from Tiangen (Cat No. DP204-03, Tiangen, Beijing, China), and the purified DNA was ligated into the pMD-19T vector (Cat No. 6013, Takara Bio, Beijing, China). This was followed by the transformation and identification of the bacterial suspension. Once confirmed, bacterial liquid samples were collected and submitted to Sangon Biotech (Shanghai, China) for sequencing [31].

### 2.5. Phylogenetic and Genomic Analysis

For the genetic analysis of the identified PRV strains, we utilized the SeqMan software within the DNASTAR platform (7.1.0.44) to assemble the sequences. These sequences were then compared with the *gB*, *gC*, *gE*, and *TK* genes of the PRV reference strains obtained from GenBank, as detailed in Table 2, using the Megalign software (7.1.0.44). To explore the genetic evolution, the MEGA11 software was utilized to construct a phylogenetic tree. The tree was generated using the neighbor-joining method, validated with a bootstrap value of 1000 replicates, and the p-distance substitution model was applied to illustrate the genetic relationships among the strains.

## 3. Results

### 3.1. Prevalence Analysis of PRV in Southwest China’s Pig Populations

In a pig farm experiencing sow abortions, we conducted an ELISA-based analysis on 469 serum samples. We found that 325 samples tested positive for PRV gE antibodies, yielding an overall positivity rate of 69.30%. Notably, the infection rate varied among different pig populations: breeding pigs had the lowest positive rate at 1.11% (1/9); suckling piglets at 53.03% (35/66); nursery pigs at 54.35% (25/46); nursing sows at 75.00% (60/80); and pregnant sows exhibited the highest rate of 76.12% (204/268), as detailed in Table 3. Additionally, the PCR-based analysis revealed that three out of six CSF samples were positive for the PRV *gE* gene, resulting in an antigen positivity rate of 50%.

To extend our investigation to additional pig farms across Southwest China, we conducted a detailed analysis of samples collected in 2022, comprising 3757 serum samples and 283 CSF samples. We identified 209 serum samples positive for PRV gE antibodies, resulting in a positivity rate of 5.56%. Moreover, three tissue samples tested positive for PRV gE antigen, yielding a positivity rate of 1.06%. Our study was further expanded to encompass the years of 2023 and 2024, during which we gathered serum and CSF samples from a variety of pig farms in the region. In 2023, 115 out of 1330 serum samples were positive for gE antibodies, with a positivity rate of 8.65%. Additionally, 7 out of 297 CSF samples were positive for gE antigen, with a positivity rate of 2.36%. In 2024, we observed a seropositivity rate of 2.36%, with 11 out of 465 serum samples testing positive (Table 4).

### 3.2. PCR Detection of PRV Antigen

**Figure 1:**
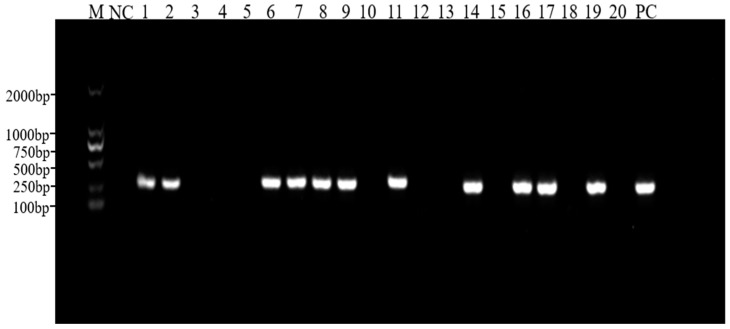
PCR amplification of the PRV *gE* gene in clinical samples. M, DNA ladder; NC, negative control; Lanes 1–20, selected representative samples; PC, positive control.

Utilizing the specific gE1 primers, we performed PCR amplification to detect PRV antigen. The PCR yielded distinct bands of the anticipated 250 bp size in certain samples, confirming the presence of PRV *gE* gene and infection with the PRV strain (Figure 1). Following this, we conducted a comprehensive PCR amplification of the *gB*, *gC*, *gE*, and *TK* genes using a selection of samples. Eight samples were randomly chosen for this analysis. Notably, seven samples exhibited identical sequences and were collectively designated as the CQ1 strain. In contrast, the eighth sample showed a unique sequence, distinguishing it as a separate strain, named CQ2. All sequences were provided in the Supplementary Materials.

### 3.3. Variation Analysis of gB Gene of Epidemic PRV Strains

**Figure 2:**
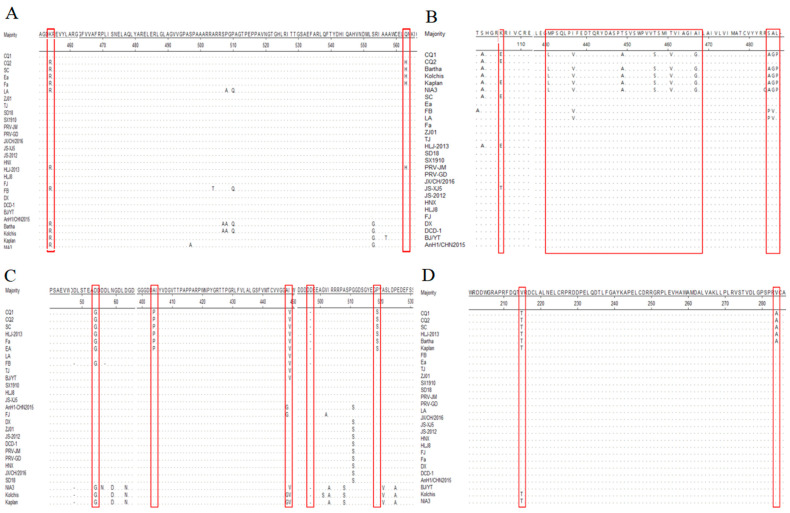
Amino acid sequence alignments of PRV gB (**A**), gC (**B**), gE (**C**), and TK (**D**). The alignment sequence is from Table 2, and the red box indicates the mutation base.

The *gB* gene sequence of PRV CQ1 strain exhibited nucleotide homology ranging from 98.2% to 99.9% when compared to both Chinese and international reference strains (Table 2). The *gB* gene of CQ2 strain showed a complete nucleotide identity with the SC strain, a Chinese classic strain isolated in 1986 [32]. A comparative analysis revealed several distinct mutations in the *gB* gene of the CQ1 strain relative to the SC strain. Specifically, a nucleotide substitution at position 1361 from guanine (G) to adenine (A) resulted in an amino acid change from Arg to Lys. Additionally, a substitution at position 1689 from G to thymine (T) led to a change from His to Glu. The remaining variable amino acid sites in the CQ1 strain’s *gB* gene were found to be identical to those of the SC strain (Figure 2A).

**Figure 3:**
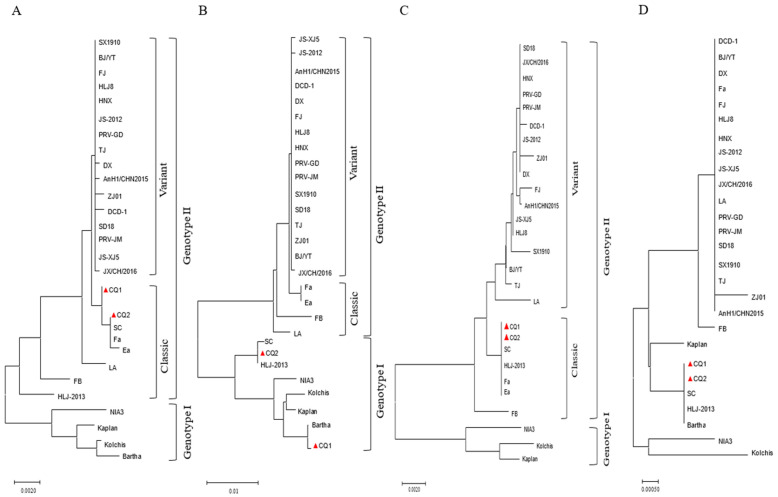
Phylogenetic tree of the *gB* nucleotide sequence (**A**), *gC* nucleotide sequence (**B**), *gE* nucleotide sequence (**C**), and *TK* nucleotide sequence (**D**). Nucleotide substitution model: p-distance; 1000 bootstrap replicates. Scale bars indicate nucleotide substitutions per site. The red triangle is the virus identified in the present study.

The phylogenetic tree analysis based on the *gB* gene categorized Chinese strains as genotype II, whereas international strains like Bartha and Kaplan were classified under genotype I (Figure 3A). Within genotype II of PRV, two distinct sub-branches were identified, which lack genetic interrelation. The CQ1 strain was found in one of these sub-branches of genotype II and showed a close genetic affinity with the CQ2 strain and other classical Chinese strains, such as SC, Fa, and Ea. These latter strains were isolated in China between the 1980s and 1990s [22,33]. Conversely, the other subcategory of genotype II encompassed Chinese variant strains such as TJ and JS-2012 that emerged after 2011 [34,35].

### 3.4. Variation Analysis of gC Gene of Epidemic PRV Strains

The *gC* gene sequence of the CQ1 strain exhibited a nucleotide homology ranging from 95.4% to 99.9% when compared to the reference strains (Table 2). Notably, the *gC* gene of the CQ1 strain exhibited the highest genetic similarity with the Bartha strain. Specifically, only a nucleotide change at position 316 from A to G resulted in an amino acid substitution from Lys to Glu. In contrast, the *gC* gene of the CQ2 strain showed complete nucleotide identity with that of the SC strain. Furthermore, the C-terminus of CQ1’s *gC* gene is the primary distinction from the CQ2 strain, with nine distinct amino acid differences. These include substitutions such as ^431^Met to Leu, ^437^Ile to Val, ^449^Thr to Ala, ^457^Thr to Ser, ^460^Ile to Val, ^467^Ala to Gly, and ^485^Ser-Ala-Leu to Arg-Gly-Pro (Figure 2B).

The phylogenetic tree analysis based on the *gC* gene categorized the PRV strains into two genotypes: genotype I and genotype II. The Chinese strains of PRV were grouped into genotype II, whereas genotype I was primarily composed of international strains (Figure 3B). Interestingly, within genotype I, the CQ1 and CQ2 strains were situated on separate sub-branches. The CQ1 strain was closely related to the Bartha strain on the same sub-branch. In contrast, the CQ2 strain was part of a sub-branch that also included the SC strain and *HLJ-2013* strain, which has been circulating since 2013 [36].

### 3.5. Variation Analysis of gE Gene of Epidemic PRV Strains

The *gE* gene sequences of CQ1 and CQ2 strains were found to be identical, showing 100% similarity with the Chinese classic strains SC and *Ea*. A comparison of the nucleotide sequence with other Chinese and international reference strains revealed a high degree of similarity, ranging from 97.4% to 100%. In contrast to the CQ1 and CQ2 strains, the Chinese variant strains contained several mutations. Specifically, a nucleotide change at position 161 from G to A resulted in an amino acid transition from Gly to Asp. Additionally, a base alteration at position 1210 from C to G led to an amino acid substitution from Pro to Ala. At position 1345, a change from G to A substituted Val with Ile. An insertion of the nucleotide sequence CGA at positions 1491–1493 introduced a new amino acid Asp at position 497. Furthermore, a nucleotide change at position 1555 from T to C resulted in the substitution of the amino acid Ser with Pro (Figure 2C).

The phylogenetic tree analysis based on the *gE* gene delineated the PRV strains into two distinct genotypes: genotype I and genotype II. Genotype I predominantly comprised international strains such as Kaplan and *Kolchis*, while genotype II was primarily composed of Chinese strains (Figure 3C). This categorization is similar to the genetic evolution tree based on the *gB* gene. Within genotype II of PRV, there were two distinct sub-branches. The CQ1 and CQ2 strains were found to cluster together with the classical Chinese strains SC, *Fa*, *Ea*, and *HLJ-2013*. This group of strains formed one of the sub-branches. The other sub-branch mainly encompassed Chinese variant strains such as *TJ*, *JS-2012*, and others.

### 3.6. Variation Analysis of TK Gene of Epidemic PRV Strains

The *TK* gene sequences of the CQ1 and CQ2 strains were identical, showing 100% homology with the SC and *HLJ-2013* strains, as well as with the Bartha strain. However, they did not share this level of homology with other classical Chinese strains, such as the *Fa* strain. The *TK* gene of the CQ1 and CQ2 strains exhibited nucleotide homology ranging from 99.5% to 100% with other reference strains, including both Chinese and international strains. A detailed comparative analysis with Chinese variant strains identified specific variations within the CQ1 and CQ2 strains. Notably, at positions 643 and 644, the GT nucleotide sequence was replaced by AC, leading to an amino acid change from Val to Thr. Additionally, at position 851, the replacement with C for T resulted in the substitution of Ala with Thr (Figure 2D).

The *TK* gene of PRV was more conserved than the *gE*, *gB*, and *gC* genes, making it more challenging to cluster based on the phylogenetic tree. For this reason, the *TK* genotype was not indicated in Figure 3D. Furthermore, the phylogenetic tree analysis confirmed a close relationship between the CQ1 and CQ2 strains and the Bartha and SC strains, indicating a strong evolutionary link (Figure 3D).

## 4. Discussion

### 4.1. Prevalence and Economic Impact of PRV Infections in Southwest China: Comparative Analysis and the Imperative for Enhanced Surveillance

Since the second half of 2011, PRV variant strains have been spreading throughout China’s pig farms, resulting in massive economic losses for the nation’s swine industry [29,37,38]. In 2022, a significant sow abortion event occurred at a large-scale pig farm in Chongqing, China. Serological analysis revealed a remarkably high average prevalence rate of PRV on the farm, reaching 69.30%. Particularly, pregnant sows exhibited the highest infection rate. However, the latest data on the seroprevalence of PRV gE antibodies at the individual animal level in China showed a relatively lower rate of 12.36% [39]. Despite this, the high positivity rate observed in our study has raised serious concerns regarding the potential for a widespread PRV epidemic across Southwest China.

To validate this perspective, we conducted an extensive collection of serum and CSF samples from large-scale pig farms across Southwest China from 2022 to 2024. Our research indicates that, in this region, the overall positive rate of PRV has been generally at a low level. This finding is corroborated by previous studies. A study by Sun Y et al. [40], conducted across 27 provinces in China from 2012 to 2017, reported an average positive rate of 8.27% among the samples tested. Similarly, Zhou H et al. [41] identified a 16.3% positive rate for PRV gE antibodies in serum samples from large-scale pig farms in Heilongjiang Province, Northeast China, during the period from 2013 to 2018. Additionally, Lin Y et al. [42] found a 23.55% incidence of positive cases in vaccinated pig farms in Hunan Province, Central China, from 2016 to 2020. These studies collectively indicate that the prevalence of PRV in Southwest China is comparable to the national average, providing a broader context for understanding the regional epidemiology of PRV infection.

Our research shows that PRV infections, leading to abortions at local pig farms, are still prevalent despite vaccinations. The virus might enter farms through various routes, including infected pigs, contaminated equipment, vehicles, or wildlife [17,43]. It might also be introduced by workers’ clothing or footwear [44]. Once inside, this virus can quickly spread in the close quarters of a pig farm, especially where biosecurity is lax. This underscores the importance of stringent surveillance and biosecurity to prevent PRV outbreaks. The differing infection rates likely result from varying farm management practices and control strategies.

### 4.2. Molecular Characterization of PRV Strains in Southwest China: Implications for Vaccine Efficacy and Disease Management

Previous studies have highlighted the diversity of PRV strains across China [14,41,42,45]. Our survey reinforces this notion, indicating that the prevalent outbreak in Southwest China is largely attributed to the Chinese classical strains classified under genotype II. Through conducting a sequence alignment analysis of the *gB*, *gC*, *gE*, and *TK* genes from the field samples, we identified two distinct PRV strains, CQ1 and CQ2. Notably, the *gE* and *gB* genes of CQ1 resemble to the domestic classic strain SC, whereas its *gC* gene displays a high degree of similarity to the Bartha strain. This genetic pattern suggests the potential occurrence of recombination events during the PRV pandemic, as reported by multiply research groups [46,47,48]. Although these four genes’ sequences of the CQ2 strain were found to be identical to those of the SC strain, current PRV vaccines, which are effective against classical strains, have been found lacking in preventing infections in local pig farms. This suggests that the existing vaccines may not be fully effective against the new recombinant forms of PRV. These findings underscore the need for a re-evaluation of current vaccination strategies and the development of more comprehensive and adaptive vaccines capable of addressing the evolving genetic landscape of PRV.

The further sequencing analysis of the CQ1 strain has revealed mutations within the *gB* and *gC* genes. The *gB* gene, which encodes an envelope glycoprotein crucial for PRV fusion with host cell membrane during infection [49], has mutations similar to those in Chinese variant strains. These genetic variations in the *gB* gene are known to influence the immunogenicity of PRV [50], suggesting that they might contribute to the increased virulence of PRV and its ability to evade the immune defenses induced by the vaccine strain Bartha [51]. The *gC* facilitates the initial attachment of PRV to the cell surface [52]. The *gC* gene of the CQ1 strain exhibits 99.9% similarity to that of the Bartha strain, with only minor nucleotide discrepancy. This high degree of genetic similarity, along with the mutations in the *gB* gene, suggests that the CQ1 strain might be a recombinant of both classical and vaccine strains, aligning with the previous reports [53,54]. This hypothesis is further supported by the resemblance of its *TK*, *gB*, and *gE* genes to those of the SC strain. In contrast, the *gB*, *gC*, *gE*, and *TK* genes of CQ2 exhibit complete homology with the corresponding genes of the SC strain, which was prevalent in the 1980s and 1990s [55]. However, it is challenging to conclusively determine whether the CQ2 strain is the original SC strain due to limitations in sample size, which precluded obtaining the full-length sequence. This constraint prevents a comprehensive genetic analysis and necessitates further investigation. Our findings suggest a rich and intricate evolutionary history of PRV strains, which could have profound effects on the efficacy of current vaccines and virus containment strategies. While the high genetic similarity between CQ2 and the historical SC strain is intriguing, the complete genetic profile and evolutionary path of CQ2 still need to be delineated.

Our survey indicates that the SC strain is not the only variant present in Southwest China, as other wild strains are also circulating. This finding adds to the already complex epidemiological scenario of PRV, emphasizing the critical need for sustained and vigilant surveillance by local pig farmers. Such vigilance is essential for gathering insights that will guide the development of effective disease management strategies tailored to the dynamic genetic landscape of PRV in the region.

## 5. Conclusions

Our findings suggest that the prevalence of PRV in the region is relatively low, with the circulating strains identified as the classical strains. However, serological data indicate that, despite extensive vaccination efforts on large-scale farms with the current PRV vaccine, these farms remain vulnerable to wild PRV infections. This implies that the existing PRV vaccine may exhibit limited efficacy against the circulating epidemic strains. In light of these findings, there is an urgent need to develop novel vaccines specifically targeting the epidemic strains. Additionally, it is crucial to strengthen PRV surveillance on pig farms, enforce epidemic prevention measures, and work systematically toward the local eradication of PRV through ongoing serological monitoring and detection.

## Figures and Tables

**Table 1:** Primers used for PCR and sequencing. Primer names indicate approximate binding positions in the PRV genome.

Primer	Sequence (5′-3′)	Amplicon Size (bp)	Primer Function
gE1-F	GCGGACGCACATGCTCTCTC	250	Detection of *gE* gene
gE1-R	CGGTCACGCCATAGTTGGGT		
gE2-F	CGTCCCCCAGCCCAAGAT	2048	Cloning PRV *gE* gene
gE2-R	GTCCCTTGGGGGCCAGCA		
gB1-F	AGACGTGCGATCAACGGCAT	1146	Segmented cloning of PRV *gB* gene
gB1-R	AACAAGGACCGCACCCTGTG		
gB2-F	ACCCGCCGCCCAGCTTAAAG	1346	
gB2-R	CGTCTCCAAGGCCGAGTACG		
gB3-F	GCAGGCCGTAGAAGGGGGAC	1126	
gB3-R	CGGCTTCTACCGCTTCCAGA		
gC-F	CGTTTCCTGATTCACGCCCAC	1917	Cloning PRV *gC* gene
gC-R	GCACCATCGACGCCAGCTC		
TK-F	GCGCACCCCGAGGTTGACTT	1255	Cloning PRV *TK* gene
TK-R	GACGGGCACGGCAAACTTT		

**Table 2:** PRV nucleotide sequences for phylogenetic analysis and the detection of PRV strains’ (CQ1 and CQ2) nucleotide (nt) and amino acid (aa) sequence identities. All sequences were obtained from the NCBI database.

					CQ1 Nucleotide (nt) and Amino Acid (aa) Sequence Identity%								CQ2 Nucleotide (nt) and Amino Acid (aa) Sequence Identity%							
					gB		gC		gE		TK		gB		gC		gE		TK	
No	GenBank	Strain	Collection Data	Region	nt	aa	nt	aa	nt	aa	nt	aa	nt	aa	nt	aa	nt	aa	nt	aa
1	MK618718.1	AnH1/CHN2015	2015	China	99.9	99.5	95.6	92.7	99.1	99.0	99.7	99.1	99.8	99.3	95.5	93.2	99.1	99.0	99.7	99.1
2	JF797217.1	Bartha	1961	Hungary	98.2	95.7	99.9	99.6	/	/	100.0	99.7	97.8	96.2	98.9	98.0	/	/	100.0	99.7
3	KC981239.1	BJ/YT	2013	China	99.9	99.6	95.6	92.7	99.6	99.5	99.7	99.1	99.8	99.5	95.5	93.2	99.6	99.5	99.7	99.1
4	OL639029.1	DCD-1	2017	China	99.8	99.5	95.6	92.7	99.1	99.0	99.7	99.1	99.7	99.3	95.5	93.2	99.1	99.0	99.7	99.1
5	MZ063026.1	DX	2012	China	99.9	99.5	95.6	92.7	99.5	99.1	99.7	99.1	99.8	99.3	95.5	93.2	99.5	99.1	99.7	99.1
6	KU315430.1	Ea	1990	China	99.9	99.5	95.6	92.5	100.0	100.0	99.5	99.1	99.9	99.8	95.5	93.0	100.0	100.0	99.5	99.1
7	ON005002.1	FB	1986	China	99.2	97.9	95.4	92.1	98.9	99.1	99.6	98.8	98.9	98.0	94.8	91.8	98.9	99.1	99.6	98.8
8	KM189913.1	Fa	2001	China	99.9	99.7	95.6	92.5	100.0	100.0	99.7	99.1	100.0	100.0	95.2	93.0	100.0	100.0	99.7	99.1
9	MW286330.1	FJ	2019	China	99.9	99.6	95.6	92.7	99.4	99.0	99.7	99.1	99.8	99.5	95.5	93.2	99.4	99.0	99.7	99.1
10	KT824771.1	HLJ8	2013	China	99.9	99.6	95.6	92.7	99.5	99.3	99.7	99.1	99.8	99.5	95.5	93.2	99.5	99.3	99.7	99.1
11	MK080279.1	HLJ-2013	2013	China	99.1	97.0	97.4	98.1	100.0	100.0	100.0	99.7	98.8	97.7	99.9	100.0	100.0	100.0	100.0	99.7
12	KM189912.1	HNX	2012	China	99.9	99.6	95.6	92.7	99.5	99.1	99.7	99.1	99.8	99.5	95.5	93.0	99.5	99.1	99.7	99.1
13	KP257591.1	JS-2012	2012	China	99.9	99.6	95.6	92.5	99.5	99.1	99.7	99.1	99.8	99.5	95.4	93.2	99.5	99.1	99.7	99.1
14	OP512542.1	JS-XJ5	2015	China	99.9	99.6	95.6	92.7	99.5	99.3	99.7	99.1	99.8	99.5	95.4	93.2	99.5	99.3	99.7	99.1
15	MK806387.1	JX/CH/2016	2016	China	99.9	99.5	95.6	92.5	99.3	99.1	99.7	99.1	99.8	99.3	95.4	93.0	99.3	99.1	99.7	99.1
16	JF797218.1	Kaplan	1987	Hungary	98.5	96.2	97.9	99.2	97.5	96.2	99.8	99.4	98.0	96.6	98.5	97.3	97.5	96.2	99.8	99.4
17	KT983811.1	Kolchis	2010	Greece	98.4	96.0	97.5	98.1	97.4	96.2	99.5	98.8	98.0	96.4	98.1	96.3	97.4	96.2	99.5	98.8
18	KU552118.1	LA	1997	China	99.6	98.8	95.7	93.1	99.3	99.5	99.7	99.1	99.6	98.9	93.9	92.0	99.3	99.5	99.7	99.1
19	KU900059.1	NIA3	1973	Belgium	98.4	96.6	97.2	97.5	97.5	95.8	99.6	99.1	98.3	96.9	97.7	95.7	97.5	95.8	99.6	99.1
20	OK338076.1	PRV-GD	2021	China	99.9	99.6	95.6	92.7	99.5	99.1	99.7	99.1	99.8	99.5	95.5	93.2	99.5	99.1	99.7	99.1
21	OK338077.1	PRV-JM	2021	China	99.9	99.6	95.6	92.7	99.5	99.1	99.7	99.1	99.8	99.5	95.5	93.2	99.5	99.1	99.7	99.1
22	KT809429.1	SC	1986	China	99.9	99.7	97.5	98.1	100.0	100.0	100.0	99.7	100.0	100.0	100.0	100.0	100.0	100.0	100.0	100.0
23	MT949536.1	SD18	2020	China	99.9	99.6	95.6	92.7	99.3	98.8	99.7	99.1	99.8	99.5	95.5	93.2	99.3	98.8	99.7	99.1
24	OL606749.1	SX1910	2022	China	99.9	99.2	95.6	92.7	99.5	99.1	99.7	99.1	99.7	99.3	95.5	93.2	99.5	99.1	99.7	99.1
25	KJ789182.1	TJ	2012	China	99.9	99.6	95.6	92.7	99.5	99.3	99.7	99.1	99.8	99.5	95.5	93.2	99.5	99.3	99.7	99.1
26	KM061380.1	ZJ01	2012	China	99.8	99.5	95.6	92.7	98.5	98.4	99.6	99.1	99.7	99.3	95.5	93.2	98.5	98.4	99.6	99.1

**Table 3:** Seroprevalence of PRV gE antibody positive rates among pigs of various ages.

Swine Herd	PRV gE Antibodies		
	Number of Detections	Positive Number	Positive Rate (%)
breeding pig	9	1	1.11%
suckling pig	66	35	53.03%
nursery pig	46	25	54.35%
nursing sow	80	60	75.00%
pregnant sow	268	204	76.12%
total	469	325	69.30%

**Table 4:** Seroprevalence and antigen detection rates of PRV gE in Southwest China from 2022 to 2024.

Year	PRV gE Antibodies			PRV gE Antigens		
	Number of Detections	Positive Number	Positive Rate (%)	Number of Detections	Positive Number	Positive Rate (%)
2022	3757	209	5.56%	283	3	1.06%
2023	1330	115	8.65%	297	7	2.36%
2024	465	11	2.36%	/	/	/

## Data Availability

The raw data supporting the conclusions of this article will be made available by the authors upon request.

## Supplementary Materials

The following supporting information can be downloaded at: https://www.mdpi.com/article/10.3390/ani14213103/s1. The sequences of this study were deposited in GenBank with the accession numbers PQ232108-PQ232115. The sequences will be released to public databases when the data or accession numbers appear in print. The sequence data are supplied in the Supplementary Files.
