# Supplementary material for: Prevalence and Genetic Variation Investigation of the Pseudorabies Virus in Southwest China
Source: Animals (Basel). 2024 Oct 28;14(21):3103. doi: 10.3390/ani14213103 (PMC11544765; doi:10.3390/ani14213103)
Supplement: Supplementary file 1 [file animals-14-03103-s001.zip › animals-3272077-supplementary.pdf]

BankIt：2863124

Seq1 GenBank accession numbers：PQ232108 CQ1/CH/2022 gB gene

1 atgcccgctg gtggcggtct ttggcgcggg ccccgcgggc atcggcccgg gcaccacggc
61 ggtgctggcc tcggacgtct ttggcctgct ccacaccacg ctgcagctgc gcgggggcgcc
121 gtcgcgctag cgctgctgct gctggcgctc gccgcgaccc cgacgtgcgg cgcggcggcc
181 gtgacgcggg ccgcctcggc ctcgcccgcg cccgggacgg gcgccacccc agacggcttc
241 tccacggagg agtccctcga ggagatcgac ggggccgtct cccccggccc ctcggacgcc
301 cccgacggcg agtacggcga cctggacgcg cgcacggccg tgcgcgcggc cgcgaccgag
361 cgggaccgct tctacgtctg cccgccgccg tccggctcca cggtggtgcg cctggagccc
421 gagcaggcct gccccgagta ctcgcagggg cgcaacttca cggaggggat cgccgtgctc
481 ttcaaggaga acatcgcccc gcacaagttc aaggcccaca tctactacaa gaacgtcatc
541 gtcacgaccg tgtggtccgg gagcacgtac gcggccatca cgaaccgctt cacggaccgc
601 gtgcccgtcc ccgtgcagga gatcacggac gtgatcgacc gccgcggcaa gtgcgtctcc
661 aaggccgagt acgtgcgcaa caaccacaag gtgaccgcct tcgaccgcga cgagaacccc
721 gtcgaggtgg acctgcgccc ctcgcgcctg aacgcgctcg gcacccgcgg ctggcacacc
781 accaacgaca cctacaccaa gatcggcgcc gcgggcttct accacacggg cacctccgtc
841 aactgcatcg tcgaggaggt ggaggcgcgc tccgtgtacc cctacgactc cttcgccctg
901 tccacggggg acatcgtgta catgtccccc ttctacggcc tgcgcgaggg ggcccacggg
961 gagcacatcg gctacgcgcc cgggcgcttc cagcaggtgg agcactacta ccccatcgac
1021 ctggactcgc gcctccgcgc ctccgagagc gtgacgcgca actttctgcg cacgccgcac
1081 ttcacggtgg cctgggactg ggcccccaag acgcggcgcg tgtgcagcct ggccaagtgg
1141 cgcgaggccg aggagatgat ccgcgacgag acgcgcgacg ggtccttccg cttcacgtcg
1201 cgggccctgg gcgcctcctt cgtcagcgac gtcacgcagc tcgacctgca gcgcgtgcac
1261 ctgggcgact gcgtcctccg cgaggcctcg gaggccatcg acgccatcta ccggcggcgc
1321 tacaacaaca cgcacgtgct ggccggcgac aagcccgagg tgtacctcgc ccgcgggggc
1381 ttcgtggtgg ccttccgccc gctgatctcg aacgagctgg cgcagctgta cgcgcgcgag
1441 ctcgagcgcc tcggcctcgc cggcgtcgtg ggccccgcgt cccccgcggc cgcccgtcgg
1501 gcccggcgct cccccggccc ggcggggacg cccgagccgc cggccgtcaa cggcacgggg
1561 cacctgcgca tcaccacggg ctcggccgag tttgcgcgcc tgcagttcac ctacgaccac
1621 atccaggcgc acgtgaacga catgctgagc cgcatcgcgg ccgcctggtg cgagctgcag
1681 aacaaggacc gcaccctgtg gggcgagatg tcgcgcctga accccagcgc cgtggccacg
1741 gccgcgctgg gccagcgcgt ctcggcgcgc atgctcggcg acgtgatggc catctcgcgg
1801 tgcgtggagg tgcgcggcgg cgtgtacgtg cagaactcca tgcgcgtgcc cggcgagcgc
1861 ggcacgtgct acagccgccc gctggtgacc ttcgagcaca acggcacggg cgtgatcgag
1921 ggccagctcg gcgacgacaa cgagctcctc atctcgcgcg acctcatcga gccctgcacc
1981 ggcaaccacc ggcgctactt taagctgggc ggcgggtacg tgtactacga ggactacagc
2041 tacgtgcgca tggtggaggt gcccgagacg atcagcacgc gggtgaccct gaacctgacg
2101 ctgctcgagg accgcgagtt cctgcccctc gaggtgtaca cgcgcgagga gctcgccgac
2161 acgggcctcc tggactacag cgagatccag cgccgcaacc agctgcacac gctcaagttc
2221 tacgacattg accgcgtggt caaggtggac cacaacgtgg tgctgctgcg cggcatcgcc
2281 aacttcttcc agggcctcgg cgacgtgggc gccgccgtcg gcaaggtggt cctgggcgcc
2341 acgggggccg tgatctcggc cgtcggcggc atggtgtcct tcctgtccaa ccccttcggg
2401 gcgctcgcca tcgggctgct ggtgctggcc ggcctggtcg cggccttcct ggcctaccgg
2461 cacatctcgc gcctgcgccg caaccccatg aaggccctgt accccgtcac gacgaaggcg
2521 ctcaaggagg acggcgtcga agaggacgac gtggacgagg ccaagctgga ccaggcccgg
2581 gacatgatcc ggtacatgtc catcgtgtcg gccctcgagc agcaggagca caaggcgcgc
2641 aagaagaaca gcgggcccgc gctgctggcc agccgcgtcg gggtgatggc cacgcgccgc

2701 cggcactacc agcgcctcga gaacgaggac cccgacgccc cctag

Seq2 GenBank accession numbers：PQ232109 CQ1/CH/2022 gC gene

1 atggcctcgc tcgcgcgtgc gatgctcgct ctgctggcgc cctacgcggc ggccatcgcc
61 gcggcgccgt cgaccacgac ggcgctcggc acgacgccca acgggggcgg cggcggcaac
121 agcagcgcgg gcgaactctc gccctcgccg cccccgaccc ccgcgcccgc ctcgcccgag
181 gcgggcgcgg tctccacgcc ccgggccccg ccgccctcgg tctcgcgcag gaagcccccg
241 cggaacaaca accggacgcg cgtccacggc gacaaggcca ccgcgcacgg gcgcgagcgc
301 atcgtgtgcc gggagcggct gttctcggcg cgggtggggg acgcggtcag cttcgggtgc
361 gccgtcttcc cgcgcgccgg ggagaccttc gaggtccgct tctaccgccg cgggcgcttc
421 cgctcgcccg acgccgaccc cgagtacttt gacgagcccc cgcggtcgga gctcccgcgg
481 gagcggctcc tcttcagctc cgccaacgcc tccctcgccc acgcggacgc gctcaccccc
541 gtcgtcgagg acgagggcgg gcgcgcgacc gtcgccaacg tctcgggcga ggtgtccgtg
601 cgcgtggccg cggcggacgc cgagaccgag ggcgtctaca cgtggcgcgt gctgtccgcc
661 aacggcaccg aggtccggag cgccaacgtc tcgctcctcc tgtacagcca gcccgagttc
721 ggcctgagcg cgccacccgt cctcttcggt gagcccttcc gggcggtgtg cgtcgtccgc
781 gactactacc cgcggcgcag cgtgcgcctg cgctggttcg cggacgagca cccggtggac
841 gccgccttcg tgaccaacag caccgtggcc gacgagctcg ggcgccgcac gcgcgtctcc
901 gtggtgaacg tgacgcgcgc ggacgtcccg ggcctcgcgg ccgcggacga cgcggacgcg
961 ctcgcgccga gcctgcgctg cgaggccgtg tggtaccgcg acagcgtggc ctcgcagcgc
1021 ttctccgagg ccctgcgccc ccacgtctac cacccggcgg cagtctcggt gcgcttcgtc
1081 gagggcttcg ccgtctgcga cggcctctgc gtgcccccgg aggcgcgcct cgcctggtcc
1141 gaccacgccg ccgacaccgt ctaccacctc ggcgcctgcg cggagcaccc cggcctgctc
1201 aacgtgcgga gcgcccgccc gctgtcggac ctcgacgggc ccgtcgacta cacctgccgc
1261 ctcgagggcc tgccctcgca gctgcccgtc ttcgaggaca cgcagcgcta cgacgcctcc
1321 cccgcgtccg tgagctggcc cgtcgtgagc agcatgatcg tcgtcatcgc cggcatcggg
1381 atcctggcca tcgtgctggt catcatggcg acgtgcgtct actaccgccg ggcgggggccg
1441 tga

Seq3 GenBank accession numbers：PQ232110 CQ1/CH/2022 gE gene

1 atgcggccct ttctgctgcg cgccgcgcag ctcctggcgc tgctggccct ggcgctctcc
61 accgaggccc cgagcctctc cgccgagacg accccgggcc ccgtcaccga ggtcccgagt
121 ccctcggccg aggtctggga cgacctctcc accgaggccg gcgacgatga cctcaacggc
181 gacctcgacg gcgacgaccg ccgcgcgggc ttcggctcgg ccctcgcctc cctgagggag
241 gcgcccccgg cccatctggt gaacgtgtcc gagggcgcca acttcaccct cgacgcgcgc
301 ggcgacggcg ccgtgctggc cgggatctgg acgttcctgc ccgtccgcgg ctgcgacgcc
361 gtgtcggtga ccacggtgtg cttcgagacc gcgtgccacc cggacctggt gctgggccgc
421 gcctgcgtcc ccgaggcccc ggagatgggc atcggcgact acctgccgcc cgaggtgccg
481 cggctccggc gcgagccgcc catcgtcacc ccggagcggt ggtcgccgca cctgagcgtc
541 ctgcgggcca cgcccaacga cacgggcctc tacacgctgc acgacgcctc ggggccgcgg
601 gccgtgttct ttgtggcggt gggcgaccgg ccgcccgcgc cggcggaccc ggtgggcccc
661 gcgcgccacg agccccgctt ccacgcgctc ggcttccact cgcagctctt ctcgcccggg
721 gacacgttcg acctgatgcc gcgcgtggtc tcggacatgg gcgactcgcg cgagaacttt
781 accgccacgc tggactggta ctacgcgcgc gcgcccccgc ggtgcctgct gtactacgtg
841 tacgagccct gcatctacca cccgcgcgcg cccgagtgcc tgcgcccggt ggacccggcg
901 tgcagcttca cctcgccggc gcgcgcgcgg ctggtggcgc gccgcgcgta cgcctcgtgc
961 agcccgctgc tcggggaccg gtggctgacc gcctgcccct tcgacgcctt cggcgaggag

1021 gtgcacacga acgccaccgc ggacgagtcg gggctgtacg tgctcgtgat gacccacaac
1081 ggccacgtcg ccacctggga ctacacgctc gtcgccaccg cggccgagta cgtcacggtc
1141 atcaaggagc tgacggcccc ggcccgggcc ccgggcaccc cgtggggccc cggcggcggc
1201 gacgacccga tctacgtgga cggcgtcacg acgccggcgc cgcccgcgcg cccgtggaac
1261 ccgtacggcc ggacgacgcc cgggcggctg tttgtgctgg cgctgggctc cttcgtgatg
1321 acgtgcgtcg tcgggggggc cgtctggctc tgcgtgctgt gctcccggcg ccgggcggcc
1381 tcgcggccgt tccgggtgcc gacgcgggcg cggacgcaca tgctctctcc ggtgtacacc
1441 agcctgccca cgcacgagga ctactacgac ggcgacgacg acgacgagga ggcgggcgtc
1501 atccgccggc ggcccgcctc ccccggcgga gacagcggct acgaggggtc gtacgcgagc
1561 ctggaccccg aggacgagtt cagcagcgac gaggacgacg ggctgtacgt gcgccccgag
1621 gaggcgcccc gctccggctt cgacgtctgg ttccgcgatc cggagaaacc ggaagtgacg
1681 aatggaccca actatggcgt gaccgccaac cgcctgttga tgtcccgccc cgcttaa

Seq4 GenBank accession numbers：PQ232111 CQ1/CH/2022 TK gene

1 atgcgcatcc tccggatcta cctcgacggc gcctacggca ccggcaagag caccacggcc
61 cgggtgatgg cgctcggcgg ggcgctgtac gtgcccgagc cgatggcgta ctggcgcact
121 ctgttcgaca cggacacggt ggccggtatt tacgatgcgc agacccggaa gcagaacggc
181 agcctgagcg aggaggacgc ggccctcgtc acggcgcagc accaggccgc cttcgcgacg
241 ccgtacctgc tgctgcacac gcgcctggtc ccgctcttcg ggcccgcggt cgagggcccg
301 cccgagatga cggtcgtctt tgaccgccac ccggtggccg cgacggtgtg cttcccgctg
361 gcgcgcttca tcgtcgggga catcagcgcg gcggccttcg tgggcctggc ggccacgctg
421 cccggggagc cccccggcgg caacctggtg gtggcctcgc tggacccgga cgagcacctg
481 cggcgcctgc gcgcccgcgc gcgcgccggg gagcacgtgg acgcgcgcct gctcacggcc
541 ctgcgcaacg tctacgccat gctggtcaac acgtcgcgct acctgagctc ggggcgccgc
601 tggcgcgacg actgggggcg cgcgccgcgc ttcgaccaga ccacgcgcga ctgcctcgcg
661 ctcaacgagc tctgccgccc gcgcgacgac cccgagctcc aggacaccct cttcggcgcg
721 tacaaggcgc ccgagctctg cgaccggcgc gggcgcccgc tcgaggtgca cgcgtgggcg
781 atggacgcgc tcgtggccaa gctgctgccg ctgcgcgtct ccaccgtcga cctggggccc
841 tcgccgcgcg cctgcgccgc ggccgtggcg gcgcaggcgc gcggcatgga ggtgacggag
901 tccgcgtacg gcgaccacat ccggcagtgc gtgtgcgcct tcacgtcgga gatgggggtg
961 tga

BankIt:2863829

Seq1 GenBank accession numbers：PQ232112 CQ2/CH/2022 gB gene

1 atgcccgctg gtggcggtct ttggcgcggg ccccgcgggc atcggcccgg gcaccacggc
61 ggtgctggcc tcggacgtct ttggcctgct ccacaccacg ctgcagctgc gcggggcgcc
121 gtcgcgctag cgctgctgct gctggcgctc gccgcgaccc cgacgtgcgg cgcggcggcc
181 gtgacgcggg ccgcctcggc ctcgcccgcg cccgggacgg gcgccacccc agacggcttc
241 tccacggagg agtccctcga ggagatcgac ggggccgtct cccccggccc ctcggacgcc
301 cccgacggcg agtacggcga cctggacgcg cgcacggccg tgcgcgcggc cgcgaccgag
361 cgggaccgct tctacgtctg cccgccgccg tccggctcca cggtggtgcg cctggagccc
421 gagcaggcct gccccgagta ctcgcagggg cgcaacttca cggaggggat cgccgtgctc
481 ttcaaggaga acatcgcccc gcacaagttc aaggcccaca tctactacaa gaacgtcatc
541 gtcacgaccg tgtggtccgg gagcacgtac gcggccatca cgaaccgctt cacggaccgc
601 gtgcccgtcc ccgtgcagga gatcacggac gtgatcgacc gccgcggcaa gtgcgtctcc
661 aaggccgagt acgtgcgcaa caaccacaag gtgaccgcct tcgaccgcga cgagaacccc
721 gtcgaggtgg acctgcgccc ctcgcgcctg aacgcgctcg gcacccgcgg ctggcacacc
781 accaacgaca cctacaccaa gatcggcgcc gcgggcttct accacacggg cacctccgtc
841 aactgcatcg tcgaggaggt ggaggcgcgc tccgtgtacc cctacgactc cttcgccctg
901 tccacggggg acatcgtgta catgtccccc ttctacggcc tgcgcgaggg ggcccacggg
961 gagcacatcg gctacgcgcc cgggcgcttc cagcaggtgg agcactacta ccccatcgac
1021 ctggactcgc gcctccgcgc ctccgagagc gtgacgcgca actttctgcg cacgccgcac
1081 ttcacggtgg cctgggactg ggcccccaag acgcggcgcg tgtgcagcct ggccaagtgg
1141 cgcgaggccg aggagatgat ccgcgacgag acgcgcgacg ggtccttccg cttcacgtcg
1201 cgggccctgg gcgcctcctt cgtcagcgac gtcacgcagc tcgacctgca gcgcgtgcac
1261 ctgggcgact gcgtcctccg cgaggcctcg gaggccatcg acgccatcta ccggcggcgc
1321 tacaacaaca cgcacgtgct ggccggcgac aggcccgagg tgtacctcgc ccgcgggggc
1381 ttcgtggtgg ccttccgccc gctgatctcg aacgagctgg cgcagctgta cgcgcgcgag
1441 ctcgagcgcc tcggcctcgc cggcgtcgtg ggccccgcgt cccccgcggc cgcccgtcgg
1501 gcccggcgct cccccggccc ggcggggacg cccgagccgc cggccgtcaa cggcacgggg
1561 cacctgcgca tcaccacggg ctcggccgag tttgcgcgcc tgcagttcac ctacgaccac
1621 atccaggcgc acgtgaacga catgctgagc cgcatcgcgg ccgcctggtg cgagctgcat
1681 aacaaggacc gcaccctgtg ggcgagatg tcgcgcctga accccagcgc cgtggccacg
1741 gccgcgctgg gccagcgcgt ctcggcgcgc atgctcggcg acgtgatggc catctcgcgg
1801 tgcgtggagg tgcgcggcgg cgtgtacgtg cagaactcca tgcgcgtgcc cggcgagcgc
1861 ggcacgtgct acagccgccc gctggtgacc ttcgagcaca acggcacggg cgtgatcgag
1921 ggccagctcg gcgacgacaa cgagctcctc atctcgcgcg acctcatcga gccctgcacc
1981 ggcaaccacc ggcgctactt taagctgggc ggcgggtacg tgtactacga ggactacagc
2041 tacgtgcgca tggtggaggt gcccgagacg atcagcacgc gggtgaccct gaacctgacg
2101 ctgctcgagg accgcgagtt cctgcccctc gaggtgtaca cgcgcgagga gctcgccgac
2161 acgggcctcc tggactacag cgagatccag cgccgcaacc agctgcacac gctcaagttc
2221 tacgacattg accgcgtggt caaggtggac cacaacgtgg tgctgctgcg cggcatcgcc
2281 aacttcttcc agggcctcgg cgacgtgggc gccgccgtcg gcaaggtggt cctgggcgcc
2341 acgggggccg tgatctcggc cgtcggcggc atggtgtcct tcctgtccaa ccccttcggg
2401 gcgctcgcca tcgggctgct ggtgctggcc ggcctggtcg cggccttcct ggcctaccgg
2461 cacatctcgc gcctgcgccg caaccccatg aaggccctgt accccgtcac gacgaaggcg
2521 ctcaaggagg acggcgtcga agaggacgac gtggacgagg ccaagctgga ccaggcccgg
2581 gacatgatcc ggtacatgtc catcgtgtcg gccctcgagc agcaggagca caaggcgcgc
2641 aagaagaaca gcgggcccgc gctgctggcc agccgcgtcg ggggtgatggc cacgcgccgc

2701 cggcactacc agcgcctcga gaacgaggac cccgacgccc cctag

Seq2 GenBank accession numbers：PQ232113 CQ2/CH/2022 gC gene

1 atggcctcgc tcgcgcgtgc gatgctcgct ctgctggcgc cctacgcggc ggccatcgcc
61 gcggcgccgt cgaccacgac ggcgctcggc acgacgccca acgggggcgg cggcggcaac
121 agcagcgcgg gcgaactctc gccctcgccg cccccgaccc ccgcgcccgc ctcgcccgag
181 gcgggcgcgg tctccacgcc ccgggccccg ccgccctcgg tctcgcgcag gaagcccccg
241 cggaacaaca accggacgcg cgtccacggc gacaaggcca ccgcgcacgg gcgcgagcgc
301 atcgtgtgcc gggagcggct gttctcggcg cgggtggggg acgcggtcag cttcgggtgc
361 gccgtcttcc cgcgcgccgg ggagaccttc gaggtccgct tctaccgccg cgggcgcttc
421 cgctcgcccg acgccgaccc cgagtacttt gacgagcccc cgcggtcgga gctcccgcgg
481 gagcggctcc tcttcagctc cgccaacgcc tccctcgccc acgcggacgc gctcaccccc
541 gtcgtcgagg acgagggcgg gcgcgcgacc gtcgccaacg tctcgggcga ggtgtccgtg
601 cgcgtggccg cggcggacgc cgagaccgag ggcgtctaca cgtggcgcgt gctgtccgcc
661 aacggcaccg aggtccggag cgccaacgtc tcgctcctcc tgtacagcca gcccgagttc
721 ggcctgagcg cgccacccgt cctcttcggt gagcccttcc gggcggtgtg cgtcgtccgc
781 gactactacc cgcggcgcag cgtgcgcctg cgctggttcg cggacgagca cccggtggac
841 gccgccttcg tgaccaacag caccgtggcc gacgagctcg ggcgccgcac gcgcgtctcc
901 gtggtgaacg tgacgcgcgc ggacgtcccg ggcctcgcgg ccgcggacga cgcggacgcg
961 ctcgcgccga gcctgcgctg cgaggccgtg tggtaccgcg acagcgtggc ctcgcagcgc
1021 ttctccgagg ccctgcgccc ccacgtctac cacccggcgg cggtctcggt gcgcttcgtc
1081 gagggcttcg ccgtctgcga cggcctctgc gtgcccccgg aggcgcgcct cgcctggtcc
1141 gaccacgccg ccgacaccgt ctaccacctc ggcgcctgcg ccgagcaccc cggcctgctc
1201 aacgtgcgga gcgcccgccc gctgtcggac ctcgacgggc ccgtcgacta cacctgccgc
1261 ctcgagggca tgccctcgca gctgcccatc ttcgaggaca cgcagcgcta cgacgcctcc
1321 cccacgtccg tgagctggcc cgtcgtgacc agcatgatca ccgtcatcgc cggcatcgcc
1381 atcctagcca tcgtgctggt catcatggcg acgtgcgtct actaccgccg gtccgcgctg
1441 tga

Seq3 GenBank accession numbers：PQ232114 CQ2/CH/2022 gE gene

1 atgcggccct ttctgctgcg cgccgcgcag ctcctggcgc tgctggccct ggcgctctcc
61 accgaggccc cgagcctctc cgccgagacg accccgggcc ccgtcaccga ggtcccgagt
121 ccctcggccg aggtctggga cgacctctcc accgaggccg gcgacgatga cctcaacggc
181 gacctcgacg gcgacgaccg ccgcgcgggc ttcggctcgg ccctcgcctc cctgagggag
241 gcgcccccgg cccatctggt gaacgtgtcc gagggcgcca acttcaccct cgacgcgcgc
301 ggcgacggcg ccgtgctggc cgggatctgg acgttcctgc ccgtccgcgg ctgcgacgcc
361 gtgtcggtga ccacggtgtg cttcgagacc gcgtgccacc cggacctggt gctgggccgc
421 gcctgcgtcc ccgaggcccc ggagatgggc atcggcgact acctgccgcc cgaggtgccg
481 cggctccggc gcgagccgcc catcgtcacc ccggagcggt ggtcgccgca cctgagcgtc
541 ctgcgggcca cgcccaacga cacgggcctc tacacgctgc acgacgcctc ggggccgcgg
601 gccgtgttct ttgtggcggt gggcgaccgg ccgcccgcgc cggcggaccc ggtgggcccc
661 gcgcgccacg agccccgctt ccacgcgctc ggcttccact cgcagctctt ctcgcccggg
721 gacacgttcg acctgatgcc gcgcgtggtc tcggacatgg gcgactcgcg cgagaacttt
781 accgccacgc tggactggta ctacgcgcgc gcgcccccgc ggtgcctgct gtactacgtg
841 tacgagccct gcatctacca cccgcgcgcg cccgagtgcc tgcgcccggt ggacccggcg
901 tgcagcttca cctcgccggc gcgcgcgcgg ctggtggcgc gccgcgcgta cgcctcgtgc
961 agcccgctgc tcggggaccg gtggctgacc gcctgcccct tcgacgcctt cggcgaggag

1021 gtgcacacga acgccaccgc ggacgagtcg gggctgtacg tgctcgtgat gacccacaac
1081 ggccacgtcg ccacctggga ctacacgctc gtcgccaccg cggccgagta cgtcacggtc
1141 atcaaggagc tgacggcccc ggcccgggcc ccgggcaccc cgtggggccc cggcggcggc
1201 gacgacccga tctacgtgga cggcgtcacg acgccggcgc cgcccgcgcg cccgtggaac
1261 ccgtacggcc ggacgacgcc cgggcggctg tttgtgctgg cgctgggctc cttcgtgatg
1321 acgtgcgtcg tcgggggggc cgtctggctc tgcgtgctgt gctcccggcg ccgggcggcc
1381 tcgcggccgt tccgggtgcc gacgcgggcg cggacgcaca tgctctctcc ggtgtacacc
1441 agcctgccca cgcacgagga ctactacgac ggcgacgacg acgacgagga ggcgggcgtc
1501 atccgccggc ggcccgcctc ccccggcgga gacagcggct acgaggggtc gtacgcgagc
1561 ctggaccccg aggacgagtt cagcagcgac gaggacgacg ggctgtacgt gcgccccgag
1621 gaggcgcccc gctccggctt cgacgtctgg ttccgcgatc cggagaaacc ggaagtgacg
1681 aatggaccca actatggcgt gaccgccaac cgcctgttga tgtcccgccc cgcttaa

Seq4 GenBank accession numbers：PQ232115 CQ2/CH/2022 TK gene

1 atgcgcatcc tccggatcta cctcgacggc gcctacggca ccggcaagag caccacggcc
61 cgggtgatgg cgctcggcgg ggcgctgtac gtgcccgagc cgatggcgta ctggcgcact
121 ctgttcgaca cggacacggt ggccggtatt tacgatgcgc agacccggaa gcagaacggc
181 agcctgagcg aggaggacgc ggccctcgtc acggcgcagc accaggccgc cttcgcgacg
241 ccgtacctgc tgctgcacac gcgcctggtc ccgctcttcg ggcccgcggt cgagggcccg
301 cccgagatga cggtcgtctt tgaccgccac ccggtggccg cgacggtgtg cttcccgctg
361 gcgcgcttca tcgtcgggga catcagcgcg gcggccttcg tgggcctggc ggccacgctg
421 cccggggagc cccccggcgg caacctggtg gtggcctcgc tggacccgga cgagcacctg
481 cggcgcctgc gcgcccgcgc gcgcgccggg gagcacgtgg acgcgcgcct gctcacggcc
541 ctgcgcaacg tctacgccat gctggtcaac acgtcgcgct acctgagctc ggggcgccgc
601 tggcgcgacg actgggggcg cgcgccgcgc ttcgaccaga ccacgcgcga ctgcctcgcg
661 ctcaacgagc tctgccgccc gcgcgacgac cccgagctcc aggacaccct cttcggcgcg
721 tacaaggcgc ccgagctctg cgaccggcgc gggcgcccgc tcgaggtgca cgcgtgggcg
781 atggacgcgc tcgtggccaa gctgctgccg ctgcgcgtct ccaccgtcga cctggggccc
841 tcgccgcgcg cctgcgccgc ggccgtggcg gcgcaggcgc gcggcatgga ggtgacggag
901 tccgcgtacg gcgaccacat ccggcagtgc gtgtgcgcct tcacgtcgga gatgggggtg
961 tga
